# Brain-Derived Exosomal Proteins as Effective Biomarkers for Alzheimer’s Disease: A Systematic Review and Meta-Analysis

**DOI:** 10.3390/biom11070980

**Published:** 2021-07-03

**Authors:** Ka Young Kim, Ki Young Shin, Keun-A Chang

**Affiliations:** 1 Department of Nursing, College of Nursing, Gachon University, Incheon 21936, Korea; kykim@gachon.ac.kr; 2 Neuroscience Research Institute, Gachon University, Incheon 21565, Korea; 3 Bio-MAX Institute, Seoul National University, Seoul 08826, Korea; 4 Department of Pharmacology, College of Medicine, Gachon University, Incheon 21936, Korea; 5 Neuroscience of Health Sciences and Technology, Gachon Advanced Institute for Health Sciences and Technology (GAIHST), Gachon University, Incheon 21936, Korea

**Keywords:** Alzheimer’s disease, biomarker, extracellular vesicle, exosome, brain-derived exosomal protein

## Abstract

Alzheimer’s disease (AD), a progressive neurodegenerative disease, affects approximately 50 million people worldwide, which warrants the search for reliable new biomarkers for early diagnosis of AD. Brain-derived exosomal (BDE) proteins, which are extracellular nanovesicles released by all cell lineages of the central nervous system, have been focused as biomarkers for diagnosis, screening, prognosis prediction, and monitoring in AD. This review focused on the possibility of BDE proteins as AD biomarkers. The articles published prior to 26 January 2021 were searched in PubMed, EMBASE, Web of Science, and Cochrane Library to identify all relevant studies that reported exosome biomarkers in blood samples of patients with AD. From 342 articles, 20 studies were selected for analysis. We conducted a meta-analysis of six BDE proteins and found that levels of amyloid-β42 (standardized mean difference (SMD) = 1.534, 95% confidence interval [CI]: 0.595–2.474), total-tau (SMD = 1.224, 95% CI: 0.534–1.915), tau phosphorylated at threonine 181 (SMD = 4.038, 95% CI: 2.312-5.764), and tau phosphorylated at serine 396 (SMD = 2.511, 95% CI: 0.795–4.227) were significantly different in patients with AD compared to those in control. Whereas, those of p-tyrosine-insulin receptor substrate-1 and heat shock protein 70 did not show significant differences. This review suggested that Aβ42, t-tau, p-T181-tau, and p-S396-tau could be effective in diagnosing AD as blood biomarkers, despite the limitation in the meta-analysis based on the availability of data. Therefore, certain BDE proteins could be used as effective biomarkers for AD.

## 1. Introduction

Alzheimer’s disease (AD) is a progressive neurodegenerative disorder [1]. The prevalence of AD in people over the age of 65 years is approximately 10%, and increases to 32% in people aged 85 years, showing increasing prevalence with age [2]. The estimated number of individuals with AD dementia is 5.8 million in the US, 9.1 million in the EU member states, and 50 million worldwide [1].

The clinical diagnosis of AD is conventionally done after neuropsychological tests and exclusion of other age-related types of dementia. Although cardinal progressive symptoms can support the clinical diagnosis, a definitive diagnosis can be made only on the postmortem examination of the brain, wherein the brain must contain sufficient amyloid plaques and neurofibrillary tangles indicative of the disease [3,4]. Therefore, the current major challenge in early AD diagnosis is the lack of reliable biomarkers, which serve as measurable indicators of the biological state or pathological condition [5]. The search for AD diagnostic targets in patients is increasing in parallel to the increasing understanding of the molecular pathogenesis of AD [5,6]. Despite substantial investments in research on AD biomarkers by governments, pharmaceutical industries, and private donors, an accurate biomarker for AD remains elusive [3].

An ideal AD biomarker should meet the following criteria: (i) The ability to detect fundamental features of AD neuropathology that can be validated on autopsy; (ii) the ability to differentiate AD from other types of dementia; (iii) the ability to detect early stages of AD and differentiate the stages of AD progression to guide therapy; (iv) should be highly reliable and the methods involved in its detection must be easy to perform and inexpensive; and (v) should have a minimally invasive sample collection method [7].

Generally, fluids [8], such as blood [9], saliva [9], urine [10], and cerebrospinal fluid (CSF) [11] and brain imaging techniques, such as structural MRI [12] and PET [13] have been used to establish a disease diagnosis and predict disease outcomes. Although CSF analysis is key in AD diagnosis, the moderately invasive nature of CSF collection limits its widespread use in routine primary clinical care practice, as the procedure is rarely performed by general practitioners [14]. Moreover, a lumbar puncture is not preferred as a routine test because it can be unpleasant and cause anxiety in patients [6,15].

Blood analysis has been promising in the diagnosis, screening, prognosis prediction, and disease monitoring for AD, and blood collection is minimally invasive, allows facile sampling, and is cost- and time-efficient [8,15]. Therefore, blood biomarkers would present a significant breakthrough in the routine screening of incipient dementia in community-based clinics if they are developed and refined based on strong concordance with CSF and brain imaging diagnostic parameters [16].

It was recently reported that brain-derived exosomes are extracellular nanovesicles released by all cell lineages of the central nervous system (CNS), and they can cross the blood-brain barrier (BBB) and be detected in the peripheral blood [17]. Glial and neuronal cell populations both release extracellular vesicles (EVs) that contain cargos of proteins such as transmembrane proteins, and lipids, RNAs, mitochondrial DNA, and single-stranded and double-stranded DNA [18]. The EVs mainly comprise exosomes ranging from a diameter of 30–140 nm, microvesicles (MVs) ranging from 100–1000 nm, and apoptotic bodies ranging from 100–5000 nm [19,20,21]. Although MVs are generated from budding of the cell membrane, they might be potential diagnostic biomarkers as they can be distinguished from other exosomes by their size and the mechanisms involved in their generation [22,23]. Due to the brain-derived exosomes contain cargo from their original cells and can be isolated from the blood, recent studies have focused the potential of brain-derived exosomal (BDE) proteins as biomarkers for diagnosis, screening, prognosis3 prediction, and monitoring in AD. However, BDE and most BDE proteins isolated from BDE are typically present in relatively low concentrations in the blood. Therefore, highly sensitive methods such as single-molecule array (SIMOA, Quanterix, USA), electrochemiluminescence enzyme-linked immunosorbent assay (Meso Scale Discovery, USA), or immunomagnetic reduction (MagQu, Taiwan) should be used [6,24]. In addition, the methods of BDE protein isolation require a high level of expertise and precision, and it is necessary to establish standardized protocols for isolation and subsequent analysis of biomarkers to address reproducibility issues [24]. Although the suitability of BDE proteins as an AD biomarkers has been controversial, the utilization of BDE proteins in the peripheral blood as AD biomarkers is promising because BDE proteins have certain advantages such as reflecting physiological changes in nervous system disorders [25]. For example, neuron-derived exosomes containing specific proteins implicated in neurodegenerative diseases can be secreted from the affected neurons [26]. In particular, BDEs from Alzheimer’s patients contain Aβ and hyperphosphorylated tau, two hallmarks of AD brains [27].

Therefore, we aimed to evaluate BDE proteins in the peripheral blood as biomarkers for AD by conducting systematic reviews with meta-analyses and discuss the possibility of BDE proteins as AD biomarkers.

## 2. Materials and Methods

### 2.1. Search Strategy

This study was performed according to the guidelines provided by the Preferred Reporting Items for Systematic Reviews and Meta-Analysis. Articles published prior to January 26, 2021 were searched in PubMed, EMBASE, Web of Science, and Cochrane Library. For a comprehensive search strategy, relevant articles written in English were retrieved using the following keywords: (exosome OR exosomal) AND (Alzheimer OR AD) AND (blood OR plasma OR serum) AND (biomarker OR “bio-marker” OR “biological marker”). The reference lists of the identified articles and relevant review articles were manually searched for additional studies.

### 2.2. Study Selection

All references obtained from the PubMed, EMBASE, Web of Science, and Cochrane Library databases were imported into the EndNote X9 reference database. Duplicate articles were automatically excluded from EndNote and the studies were selected manually again. The articles were then analyzed to check their relevance in the context of our study. The abstracts of the relevant articles were then reviewed to identify eligible papers. Articles including commentaries, letters, editorials, conference abstracts, and reviews were excluded from this study. Full-text articles were then obtained and reviewed thoroughly to identify studies reporting exosomal biomarkers in the blood samples of patients with AD. The inclusion criteria for the studies were as follows: (1) Articles that compared patients with AD and healthy controls; (2) articles that identified plasma or serum vesicles; (3) articles assessing protein biomarkers in exosomes. The exclusion criteria were as follows: (1) Articles that used animal models; (2) articles that included a control group with other diseases, such as Down’s Syndrome, HIV/AIDS, and Parkinson’s disease; (3) articles not related to blood exosomes; (4) articles not related to protein biological biomarkers; (5) articles in the form of a commentary, letter, editorial, conference abstract, and review.

### 2.3. Data Extraction

Two authors (K.Y. Kim and K. Chang) independently screened and selected relevant studies according to the inclusion and exclusion criteria. All authors (K.Y. Kim, K.Y. Shin, and K. Chang) constantly discussed the articles to resolve any disagreements. The relevant data, including the first author, publication year, study country, number of participants in the control and patient groups, sex of the participants, age of the participants, and Mini-Mental State Examination (MMSE) scores were extracted from the selected studies. We classified the identified BDE proteins into related categories. Furthermore, the levels of BDE proteins in AD were analyzed.

### 2.4. Statistical Analysis

For the meta-analysis, the standardized mean difference of BDE proteins between the AD and control groups was analyzed using the Comprehensive Meta-Analysis software version 3 (Biostats Inc., Englewood, NJ, USA). Moreover, we used the Q statistic and *I*^2^ method to analyze the heterogeneity. A random-effects model was used to account for heterogeneity. Publication bias was assessed using funnel plots and Egger’s intercept test. A *p*-value of <0.05 was considered statistically significant.

## 3. Results

### 3.1. Characteristics of the Eligible Studies

**Figure 1:**
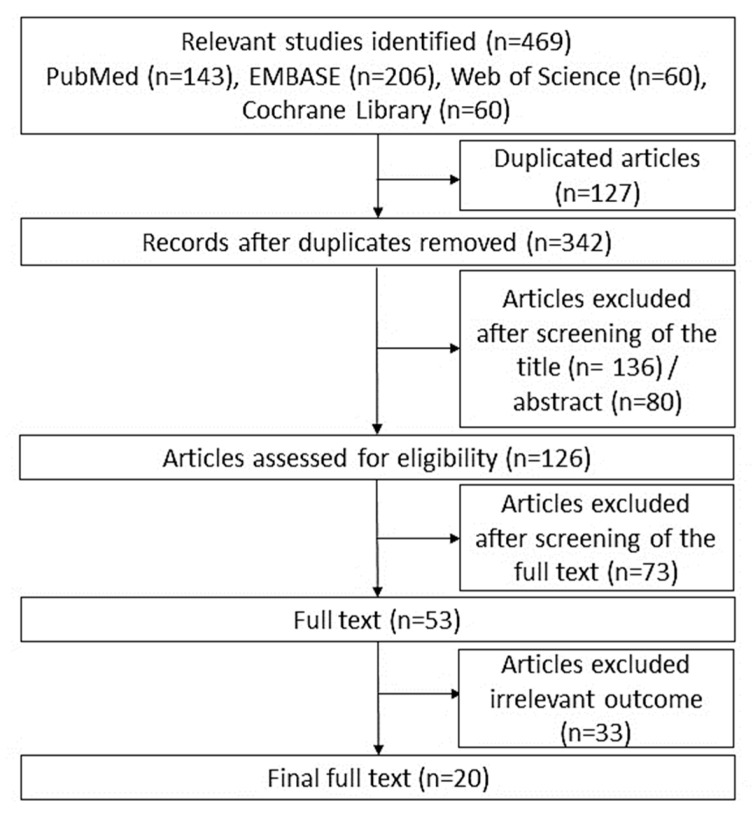
Flow chart of study selection.

Figure 1 presents the flow chart of the study selection. From the 342 articles, 20 were selected after a detailed review of the full texts of all eligible studies.

Table 1 presents the general characteristics of the 20 studies included. All the studies were published between 2015 and 2021. These studies were conducted in regions including the USA, Italy, Spain, Sweden, China, Korea, and Canada. The specimens used in the studies were plasma or serum. The samples were grouped into cases, including AD that presented original articles and controls and cases including normal or healthy individuals, with the sample size ranging from 8 to 350. The MMSE scores were presented in the AD, and control groups, respectively.

Furthermore, Table 1 shows the BDE proteins for AD used in the selected studies. The identified Aβ-targeted biomarkers were amyloid-β42 (Aβ42), amyloid precursor protein (APP), soluble APP alpha (sAPPα), sAPPβ, Aβ42/tau phosphorylated at threonine 181 (p-T181-tau), BACE-1, and γ-secretase. Tau-targeted biomarkers were total-tau (t-tau), p-T181-tau, p-T231-tau, p-S202-tau, tau phosphorylated at serine 396 (p-S396-tau), N-224 tau, N-123 tau, MR tau, FL tau, p-tau/t-tau, p-T181-tau/t-tau, and t-tau/Aβ42. The extracted synaptic protein biomarkers were synaptophysin, synaptotagmin, synaptopodin, neurogranin (NRGN), synaptosomal-associated-protein-25 (SNAP-25), GluA4-containing glutamate (AMPA4) receptor, pentraxin 2 (NPTX2), neuroligin 1 (NLGN1), neurexin 2 (NRXN2), P-S9-synapsin 1, growth-associated protein 43 (GAP43), synapsin 1, and myelin-oligodendrocyte glycoprotein (MOG). Autolysosomal proteins were cathepsin D and lysosome-associated membrane protein 1 (LAMP-1). Growth or trophic factors were fibroblast growth factors (FGF)-2, FGF-13, glial-derived neurotrophic factor (GDNF), hepatocyte growth factor (HGF), and type 1 insulin-like growth factor (IGF-1). Brain insulin resistance-related exosomal protein biomarkers were total insulin receptor substrate-1 (t-IRS-1), P-serine 312-IRS-1 (p-S312-IRS-1), P-tyrosine-IRS-1 (p-Y-IRS-1), and p-S312-IRS-1/p-Y-IRS-1. Inflammation-related exosomal protein biomarkers included interleukin 6 (IL-6), matrix metalloproteinase-9 (MMP-9), and translocator protein (TSPO). Heat-shock protein 70 (HSP70) and ubiquitinylated protein were the exosomal protein biomarkers related to molecular chaperons. Repressor element 1-silencing transcription factor (REST) was a transcriptional repressor biomarker. The cluster of differentiation 81 (CD81) and TSG101 were the cell-type marker-related BDE proteins, and exosome marker-related BDE proteins were glial fibrillary acidic protein (GFAP), glutamine synthetase (GluSyn), neurofilament light chain (NF-Lch), and neuron-specific enolase (NS-enolase). Other BDE proteins were growth-associated protein 43 (GAP43), ganglioside M1 (GM1), and Septin-8.

### 3.2. BDE Protein Changes in AD

Table 2 shows the BDE protein changes in AD. Of the identified BDE proteins, the increased level in AD were Aβ42, APP, sAPPβ, BACE-1, t-tau, p-T181-tau, p-T231-tau, p-S202-tau, p-S396-tau, p-tau/t-tau, p-T181-tau/t-tau, t-tau/Aβ42, cathepsin D, LAMP-1, GDNF, p-Y-IRS-1, p-S312-IRS-1, p-S312-IRS-1/p-Y-IRS-1, MMP-9, TSPO, ubiquitinylated protein, GFAP, NF-Lch, NS-enolase, and GM1. BDE proteins that decreased in AD were Aβ42, APP, t-tau, NRGN, synaptophysin, synaptotagmin, synaptopodin, SNAP-25, AMPA4 receptor, NPTX2, NLGN1, NRXN2, P-S9-synapsin 1, synapsin 1, MOG, GDNF, FGF-2, FGF-13, HGF, IGF-1, p-Y-IRS-1, HSP70, REST, GFAP, GluSyn, CD81, GAP43, and Septin-8. The extracted BDE proteins that have no change in AD were Aβ42, APP, sAPPα, sAPPβ, BACE-1, Aβ42/p-T181-tau, γ-secretase, t-tau, p-T181-tau, p-S396-tau, p-T181-tau/t-tau, t-tau/Aβ42, N-224 tau, N-123 tau, MR tau, FL tau, cathepsin D, LAMP-1, t-IRS-1, p-Y-IRS-1, p-S312-IRS-1, p-S312-IRS-1/p-Y-IRS-1, IL-6, HSP70, ubiquitinylated protein, GluSyn, NF-Lch, NS-enolase, CD81, TSG101, and Septin-8.

**Figure 2:**
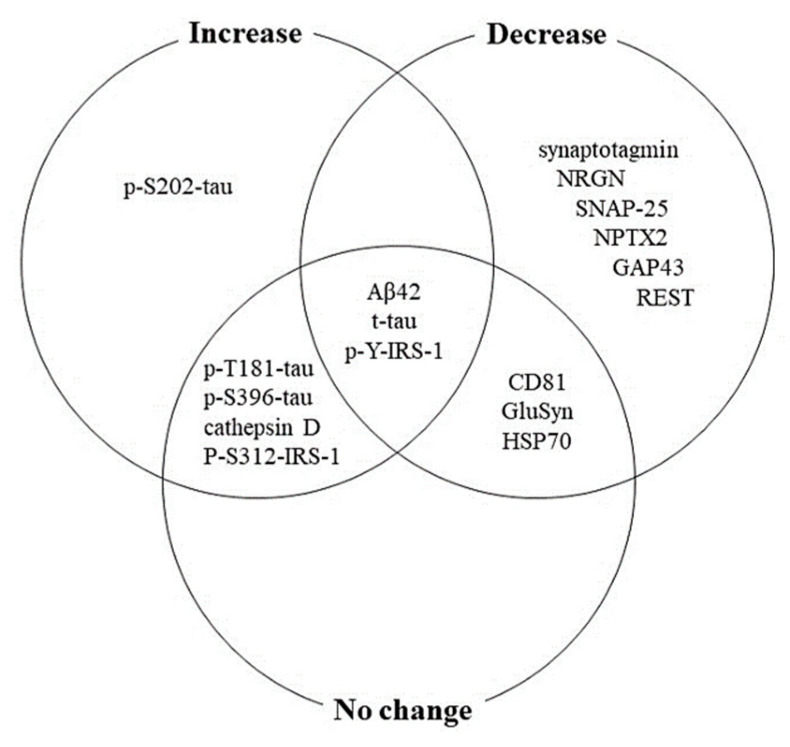
Figure showing exosomal proteins in Alzheimer’s disease (AD). This diagram represents exosome proteins that were identified in two or more articles.

The BDE proteins that were identified in two or more articles are shown in Figure 2. Aβ42, t-tau, and p-Y-IRS-1 were the BDE proteins whose levels increased or decreased or had no change in AD. The levels of BDE proteins that increased or had no change in AD were p-T181-tau, p-S396-tau, cathepsin D, and p-S312-IRS-1. CD81, GluSyn, and HSP70 were the BDE proteins whose levels decreased or had no change in AD.

### 3.3. Meta-Analysis Results of Aβ42, t-tau, p-Y-IRS-1, p-T181-tau, p-S396-tau, and HSP70

**Figure 3:**
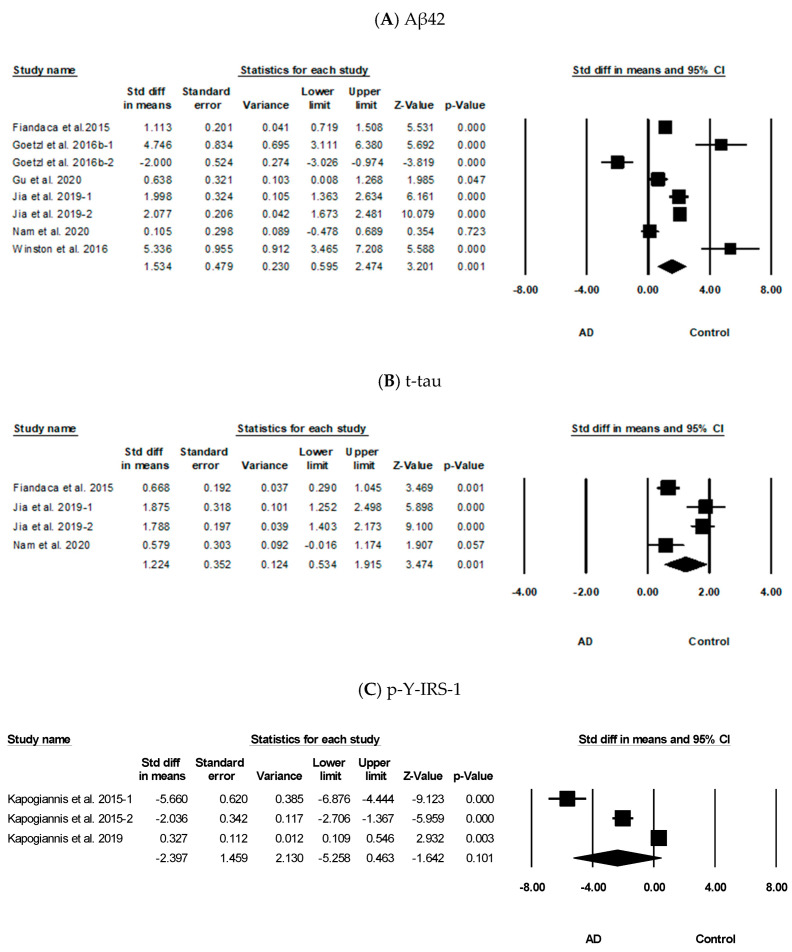

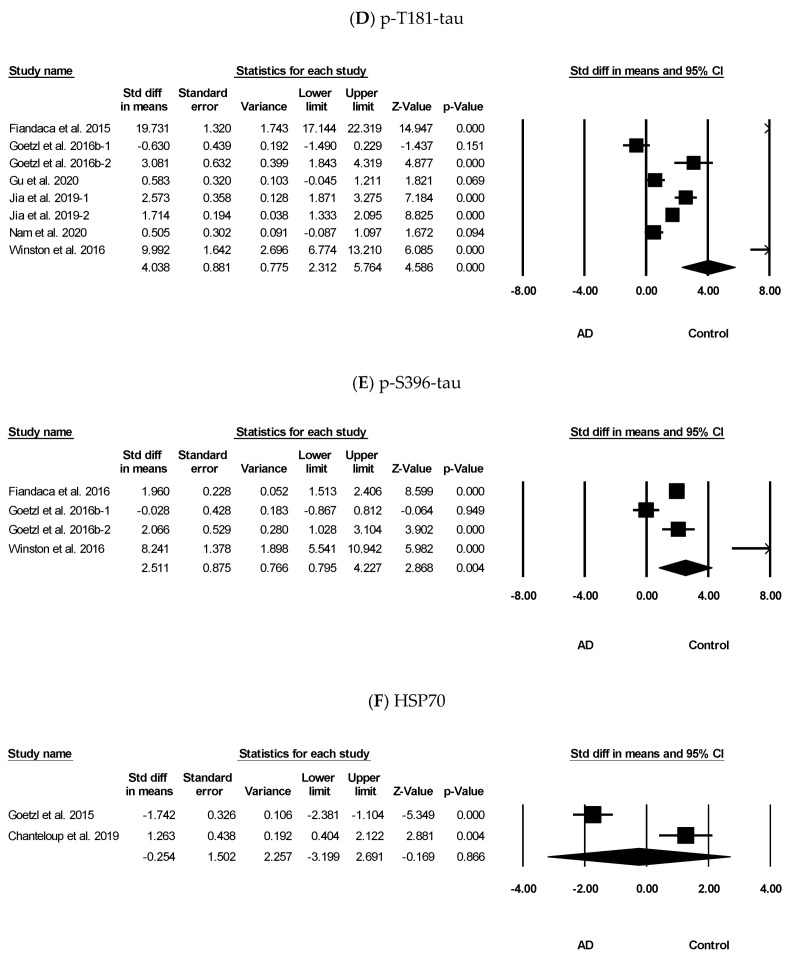
Forest plots of Aβ42, t-tau, p-Y-IRS-1, p-T181-tau, p-S396-tau, and HSP70. Effects sizes were measured as the standardized mean difference in AD sample compared to controls. (**A**) Aβ42, (**B**) t-tau, (**C**) p-Y-IRS-1, (**D**) p-T181-tau, (**E**) p-S396-tau, and (**F**) HSP70. Std diff, standard difference; CI, confidence interval.

Figure 3 shows the results of the meta-analysis of duplicated BDE proteins in AD from two or more articles. As shown in Figure 3A, the meta-analysis of Aβ42 showed that patients with AD had significantly high levels of this protein than the controls (standardized mean difference [SMD] = 1.534, 95% confidence interval [CI]: 0.595 to 2.474, *p* = 0.001). The total tau protein levels showed a significant increase in patients with AD (SMD = 1.224, 95% CI: 0.534 to 1.915, *p* = 0.001) (Figure 3B). The meta-analysis results of p-Y-IRS-1 showed that patients with AD had no significant differences in the protein levels compared with the controls (SMD = −2.397, 95% CI: −5.258 to 0.463, *p* = 0.101) (Figure 3C). Furthermore, the p-T-181-tau protein levels showed that patients with AD had significantly high protein levels (SMD = 4.038, 95% CI: 2.312 to 5.764, *p* < 0.001) (Figure 3D). As shown in Figure 3E, p-S396-tau protein levels had a significant increase in patients with AD (SMD = 2.511, 95% CI: 0.795 to 4.227, *p* = 0.004). Furthermore, HSP70 protein levels (SMD = -0.254, 95% CI: −3.199 to 2.691, *p* = 0.866) (Figure 3F) showed that there was no significant difference in the levels in patients with AD compared with that in the controls.

We used the random effect model in this study because the heterogeneity was significant (Figure 3A: *I*^2^ = 94%, *p* < 0.001; 3B: *I*^2^ = 88%, *p* < 0.001; 3C: *I*^2^ = 98%, *p* < 0.001; 3D: *I*^2^ = 97%, *p* < 0.001; 3E: *I*^2^ = 93%, *p* < 0.001; 3F: *I*^2^ = 97%, *p* < 0.001). Publication bias was evaluated using Egger’s regression test. None of the data showed an obvious risk of publication bias (Figure 3A: *p* = 0.77; 3B: *p* = 0.95; 3D: *p* = 0.10; 3E: *p* = 0.61), except for that of p-Y-IRS-1 (*p* = 0.04).

## 4. Discussion

Biomarkers for AD are of great importance since the cognitive symptoms of AD are often diffuse, and overlap with those of other disorders and the clinical progression of AD is variable even among patients with the same disease. Alzheimer’s disease is still poorly diagnosed despite the availability of numerous theoretical and clinical diagnostic tools as these tools lack specific biomarkers, have procedural and methodological inconsistencies, and insufficient standardization assays [48].

Cargos of cell-specific exosomes indicate pathological conditions and are closely associated with the stages of AD [49]. In particular, BDE proteins with enriched levels of exosomes secreted from the nervous system during AD could contribute to a more accurate AD diagnosis, and could help further discover close connections between the markers and mechanisms of the early stage of the disease [17,24]. Additionally, these BDE proteins can simultaneously reflect the pathology of the brain of patients. The relatively poor performance of blood-based biomarkers reflects the disconnection between brain biochemistry and blood composition, which is maintained by the BBB to protect the brain [24]. For example, plasma Aβ and tau levels have not mirrored the sensitivity and specificity of their CSF counterparts [16]. However, BDE proteins can cross the BBB to get into the blood and can be isolated by immunoprecipitation using antibodies specific for brain protein markers such as neural cell adhesion molecule L1 (L1CAM) and cell adhesion molecules [24]. Recent reports have shown that the levels of Aβ and tau as BDE proteins are higher in the blood of patients with AD compared with the controls and are significantly correlated with those in the CSF [37,49,50]. Therefore, the development of BDE proteins in peripheral blood as a diagnostic biomarker of AD has a great potential as they can indicate brain biochemistry in detail and mirror their CSF counterparts [28]. Therefore, we focused on BDE proteins as potential biomarkers for AD in this review because of their advantages.

We selected six BDE proteins that have inconsistent results from different experiments and analyzed whether they could be effective in AD diagnosis. As shown in Figure 3, the levels of Aβ42, t-tau, p-T181-tau, and p-S396-tau were higher in patients with AD than in the controls. Unfortunately, there were no differences between the levels of p-Y-IRS-1 and HSP70 in patients with AD and the controls. Our results suggest that Aβ42, t-tau, p-T181-tau, and p-S396-tau in BDE may be effective biomarkers, as detection strategies based on novel biomarkers, like Aβ and tau proteins could represent a promising solution for the early diagnosis of AD [8].

The two core neuropathological hallmarks of AD are Aβ and tau protein aggregates. The first hallmark is the presence of Aβ deposits in the brain parenchyma as neuritic plaques and around cerebral blood vessels as cerebral amyloid angiopathy [51,52,53]. The Aβ peptide present in amyloid plaques is approximately 36–43 amino acids in length, and is generated from APP by a series of proteolytic cleavages followed by a broad range of post-translational modifications [54]. Aβ plays a major role in neurotoxicity and neural function; therefore, accumulation of dense plaques in the hippocampus, amygdala, and cerebral cortex can cause stimulation of astrocytes and microglia, damage to the axons and dendrites, loss of synapses, and cognitive impairments [55,56,57,58]. The second hallmark is neurofibrillary tangles (NFTs) and hyperphosphorylated tau, which accumulate intracellularly and are typically accompanied by neuronal loss [51]. The tau protein is hyperphosphorylated in AD, which leads to compromised microtubules, thereby disrupting several cellular processes, such as proliferation, differentiation, protein trafficking, and cellular morphology [59,60]. NFTs are abnormal filaments of hyperphosphorylated tau proteins that can be twisted around each other in some stages to form paired helical filaments and accumulate in the neural perikaryal cytoplasm, axons, and dendrites, which causes a loss of cytoskeletal microtubules and tubulin-associated proteins [55]. However, inconsistencies between results have been reported in many studies and a lack of correlation between CSF and blood Aβ has been observed. These results were probably due to low Aβ concentrations in the blood [8,25,61,62]. Plasma t-tau concentrations also correlate poorly with that in the CSF. Assays for the quantification of tau have been hampered by a lack of analytical sensitivity and accurate measurements [6,25,63]. Our analysis also included inconsistent results (Figure 2). Both the Aβ42 and t-tau protein levels showed an increase, decrease, or no change between the control and AD groups. Hence, the levels of p-T181-tau and p-S396-tau both showed an increase or no change between the two groups. However, our meta-analysis indicated that the concentrations of Aβ42, t-tau, p-T181-tau, and p-S396-tau were higher in patients with AD than in the controls. A recent study comparing the diagnostic value of total plasma exosomes and plasma-derived BDEs showed that plasma BDEs had a more promising potential diagnostic value than plasma exosomes [64]. Our results showed that the concentrations of Aβ42, t-tau, and P-T181-tau in the AD group were higher than those in the amnestic mild cognitively impaired (aMCI) and control groups. The level of each BDE biomarker in the blood was highly correlated with that in the CSF. Therefore, this study verified the association between CSF and blood BDE biomarkers [37]. Another study showed that the levels of soluble Aβ42 and other proteins involved in the Aβ42 generating pathway are higher in astrocytic-derived exosomes than that in neuronal exosomes [33,65]. Additionally, patients with AD showed a 3–20-fold increase in p-T181-tau and p-S396-tau levels among other BDE proteins [17,66]. Moreover, the p-T181-tau levels were significantly higher in BDE proteins isolated from the plasma of patients with late-stage AD than patients with AD who had only been diagnosed with mild cognitive impairment [34]. This demonstrate a dysfunction of the clearance ability or an increase in the pathogenicity of exosomes in later stages of AD [48]. Additionally, the use of highly sensitive methods, such as SIMOA, electrochemiluminescence enzyme-linked immunosorbent assay, or immunomagnetic reduction could help detect commonly occurring low concentrations of exosomes [6,24].

Although our results of p-Y-IRS-1 and HSP70 were not significant, it is necessary that the two proteins be consider after the future research is conducted. The aforementioned proteins have the following characteristics: First, IRS-1 serves as the effector molecule of the insulin receptor [67]. Normal tissue responses to insulin include enhanced glucose uptake, altered metabolism, and changes in cellular function. A diverse range of reduced responses to insulin in the brain and peripheral tissues is designated as insulin resistance [30,68]. Brain insulin resistance is dependent on IRS-1 phosphorylation, and is important in AD pathogenesis as it may potentially be linked to amyloid and tau pathologies [67,69]. It was reported that the brain volume of patients with AD was positively associated with p-Y-IRS-1 in the exosomes isolated from plasma [67]. Interestingly, the importance of IRS-1 phosphotypes including p-Y-IRS-1 as predictive biomarkers for AD has been suggested [42]. The negative association of p-S312-IRS-1 and p-Y-IRS-1 with cognition was replicated in an in vivo study between these markers in autopsied brains of AD participants and antemortem cognition [70]. Additionally, tau hyperphosphorylation induces brain insulin resistance, and this induction may be reflected in the strong associations between p-T231-tau and p-T181-tau with p-S312-IRS-1 and p-Y-IRS-1 [42]. Second, HSPs constitute a group of highly conserved ubiquitous chaperones, which are expressed in response to several conditions. The HSP70 protein is a universal stress-inducible chaperone, and is a key regulator of proteostasis that interacts with misfolded proteins present in neurodegenerative disorders regulating aggregation or refolding and amending those that are incorrectly folded [71]. HSP70 was identified in AD as a protector against intracellular Aβ accumulation, as its overexpression rescued neurons from Aβ-mediated toxicity [72,73]. To explain this phenomenon, it has been proposed that HSP70 attenuates the cytotoxicity of Aβ by binding amyloidogenic peptides and restoring the balance between aggregation, folding, and degradation [72]. Additionally, the correlation with FDG-PET suggested that exosomal HSP70 may be a marker of the degree of synaptic failure or neurodegeneration [39]. Moreover, the levels of HSP70 were significantly lower in neural-derived plasma exosomes of patients with AD than in control plasmas [29].

In addition to the six proteins selected in our study, many proteins have been demonstrated as possible biomarkers, but we could not meta-analyze these proteins because of insufficient results. As shown in Table 2, the levels of synaptic proteins such as NRGN, synaptophysin, synaptotagmin, synaptopodin, SNAP-25, AMPA4 receptor, NPTX2, NLGN1, NRXN2, p-S9-synapsin 1, synapsin 1, and MOG were lower in patients with AD. The levels of growth factors such as FGF-2, FGF-13, HGF, and IGF-1 were also reduced in patients with AD. Additionally, the levels of REST and GAP43 decreased in patients with AD. In contrast, the levels of tau-related proteins, such as p-T231-tau, p-S202-tau, and p-tau/t-tau ratio increased in patients with AD. The levels of MMP-9, TSPO, and GM1 also increased in patients with AD. If the results for the aforementioned proteins are further collected in relevant studies, it might be certain to obtain possible biomarkers for AD.

There is an additional merit in finding specific BDE proteins that represent a novel class of therapeutic targets besides their use as biomarkers. For example, exosomes injected into the brain of transgenic mouse models of AD helped decrease toxic oligomers and fibrils in a microglial-dependent manner following intracerebral administration, contributing to the clearance of Aβ in vivo [17,74,75,76]. Exosomes derived from neurons, astrocytes, oligodendroglia, and microglia have different functions [76] such as: (i) The role of the exosomes released from neurons may be related to synaptic plasticity, neurovascular communication, neuroprotection, and neuroregeneration [77,78,79,80,81]; (ii) the role of astrocyte-derived exosomes may be associated with neuronal survival, synaptic transmission, neuroinflammation, and neurogenesis [82,83,84,85]; (iii) the role of oligodendroglia-secreted exosomes may be linked to axon development, neuronal integrity, and enhanced neuronal stress tolerance [86,87]; and (ix) the role of exosomes sourced from microglia may be correlated with neuronal survival, neurite outgrowth, and neuroinflammatory response [88,89,90]. Therefore, if cell-specific damage using BDE proteins could be detected accurately, it could provide therapeutic targets and novel drug delivery vehicles, as well as help in the diagnosis and prognosis prediction for AD. Interestingly, Yin et al. reported that exosomes have a therapeutic potential in treating AD by enhancing neuroprotection mechanisms and acting as therapeutic vehicles, and they may play a vital role in AD preclinical and clinical studies as biomarkers [91].

However, this study had certain limitations. First, we had limited results because we used data only from the papers included/selected in this study. Second, our results included the control and AD groups regardless of the stage of AD. Therefore, further research is needed to analyze the stages of AD, as well as mild cognitive impairment. Nevertheless, levels of BDE proteins including Aβ, total tau, or p-tau in patients with AD exhibit a remarkable change. Therefore, we demonstrated that BDE proteins, such as Aβ, total tau, or p-tau could be potential biomarkers for the diagnosis, prognosis prediction, and progression of AD.

## Figures and Tables

**Table 1:** Characteristics of the eligible studies.

Study	Location	Specimen	Method	Patients	Sample (M/F)		Age (y) (M ± SD)		MMSE (M ± SD)		BDE Proteins
					Case	Control	Case	Control	Case	Control	
Fiandaca et al. 2015 [28]	USA	Plasma	ExoQuick/ELISA	AD	30/27	30/27	79.5 ± 6.1	79.6 ± 6.0			Aβ42, t-tau, p-T181-tau, p-S396-tau
Goetzl et al. 2015 [29]	USA	Plasma	ExoQuick/ELISA	AD (include MCI)	13/13	13/13	75.4 ± 7.9	75.8 ± 7.9	22.5 ± 1.5		cathepsin D, LAMP-1, HSP70, ubiquitinylated protein
Kapogiannis et al. 2015 [30]	USA	Plasma	ExoQuick/ELISA	AD	13/13	13/13	74.3 ± 7.5	74.3 ± 7.5			t-IRS-1, p-S312-IRS-1, p-Y-IRS-1, p-S312-IRS-1/p-Y-IRS-1
				Dementia	10				20.5 ± 2.2		
Abner et al. 2016 [31]	USA	Plasma	ExoQuick/ELISA	AD	5/5	10/10	77.6	77.6		29.4 ± 0.8	Aβ42, p-T181-tau, NRGN, cathepsin D, REST
Goetzl et al. 2016a [32]	USA	Plasma	ExoQuick/ELISA	AD	6/6	6/6	74.4 ± 2.0	74.4 ± 2.0	26.3 ± 1.0	29.8 ± 0.1	Synaptophysin, synaptotagmin, synaptopodin, NRGN, p-S9-synapsin 1, GAP43, synapsin 1, MOG, GAP43
				AD2 (after diagnosis of dementia)	2/7	2/7	87.8 ± 2.5	82.2 ± 2.3	21.4 ± 1.6	28.3 ± 1.0	
Goetzl et al. 2016b [33]	USA	Plasma	ExoQuick/ELISA	AD (include amnestic mild cognitive impairment and early dementia)	12	10					Aβ42, sAPPα, sAPPβ, BACE-1, γ-secretase, p-T181-tau, p-S396-tau, GDNF, GFAP, GluSyn, NF-Lch, NS-enolase, CD81, Septin-8
Winston et al. 2016 [34]	USA	Plasma	ExoQuick/ELISA	AD	11/9	10	75.4 ± 6.8		17.7 ± 0.7		Aβ42, p-T181-tau, p-S396-tau, NRGN, REST
Guix et al. 2018 [35]	USA	Plasma	ExoQuick/ELISA	AD (mild)	3/7	3/7	75.6 ± 12.9	75.9 ± 8.7	75.6 ± 12.9	29.7 ± 0.5	Aβ42, p-T181-tau, MR tau, FL tau
				AD (moderate)	4/6		75.6 ± 12.9		75.6 ± 12.9		
Goetzl et al. 2018 [36]	USA	Plasma	ExoQuick/ELISA	AD	12/16	12/16	73.1 ± 1.4	73.2 ± 1.5	25.6 ± 0.8	29.7 ± 0.1	AMPA4 receptor, NPTX2, NLGN1,
				AD2 (after diagnosis of dementia)	10/8	10/8	78.2 ± 1.8	70.1 ± 1.7	20.2 ± 1.5	28.3 ± 1.0	NRXN2
Jia et al. 2019 [37]	China	Plasma	ExoQuick/ELISA	AD	39/42	35/37	65 ± 6	64 ± 5	19.6 ± 3.1	29.3 ± 1.2	Aβ42, p-T181-tau
Agliardi et al. 2019 [38]	Italy	Serum	ExoQuick/ELISA	AD	8/16	4/13	77.7 ± 1.4	76.5 ± 1.5	21.9 ± 0.9	28.7 ± 0.4	SNAP-25
Chanteloup et al. 2019 [39]	Spain	Plasma	ExoQuick/ELISA	AD	21	13	77.1 ± 8.2	75.2 ± 6.7			HSP70
Cicognola et al. 2019 [40]	Sweden	Serum	ExoQuick/SIMOA	AD	4	4	79.5	67	>15		N-224 tau, N-123 tau
Goetzl et al. 2019 [41]	USA	Plasma	ExoQuick/ELISA	AD	9/15	9/15	73.1 ± 1.6	73.1 ± 1.8	26.1 ± 0.9	29.3 ± 0.2	AMPA4 receptor, FGF-2, FGF-13, HGF, IGF-1, GluSyn, CD81
				AD2 (after conversion to moderate dementia)	7/8	7/8	84.5 ± 1.7	80.2 ± 1.8	24.3 ± 0.9	29.4 ± 0.6	
Kapogiannis et al. 2019 [42]	USA	Plasma	ExoQuick/ELISA, SIMOA	AD (future)	60/68	112/110	79.1 ± 7.0	76.2 ± 7.4	27.5 ± 1.8	28.4 ± 1.8	t-tau, p-T181-tau, p-T231-tau, p-S312-IRS-1, p-Y-IRS-1, TSG101
		Serum		AD	17/18	6/23	74.0 ± 8.7	72.1 ± 7.9	23.9 ± 3.0	29.8 ± 0.6	
Gu et al. 2020 [43]	China	Plasma	ExoQuick/ELISA	AD	8/23	5/10	68.6 ± 8.0	64.8 ± 6.0	15.9 ± 6.6	27.7 ± 1.7	Aβ42, p-T181-tau, p-S396-tau, IL-6, MMP-9, CD81
Jia et al. 2020 [44]	China	Plasma	ExoQuick/ELISA	AD	59/62	74/86	66 ± 5	54 ± 6	20.7 ± 2.9	29.1 ± 1.1	Synaptotagmin, NRGN, SNAP-25, GAP43
Nam et al. 2020 [45]	Korea	Serum	ExoQuick/ELISA	AD	3/17	17/9	76.6 ± 1.3	73.9 ± 0.9	16.6 ± 0.5	27.7 ± 0.2	Aβ42, t-tau, p-T181-tau, p-S202-tau, p-tau/t-tau
Perrotte et al. 2020 [46]	Canada	Plasma	Exosome isolation kit/Luminex	AD (mild)	1/11	3/9	75.6 ± 1.3	68.8 ± 1.5	24.0 ± 0.5	29.4 ± 0.3	Aβ42, APP, Aβ42/p-T181-tau, t-tau, p-T181-tau, p- T181-tau/t-tau, t-tau/Aβ42
				AD (moderate)	4/8		79.1 ± 1.1		19.9 ± 1.4		
				AD (severe)	2/10		83.0 ± 1.6				
Picciolini et al. 2021 [47]	Italy	Plasma	Chromatography using qEV columns/ELISA	AD	4/6	5/5	73.9 ± 3.0	62.6 ± 2.0			TSPO, GM1

AD: Alzheimer’s disease, ELISA: enzyme-linked immunosorbent assay, MCI: mild cognitive impairment, MMSE: mini-mental state examination, SIMOA: single molecule array.

**Table 2:** Changes of exosomal proteins in AD.

Category	Level	Exosomal Proteins
Aβ targeted	Increase	Aβ42, APP, sAPPβ, BACE-1
	Decrease	Aβ42, APP
	No change	Aβ42, APP, sAPPα, sAPPβ, BACE-1, Aβ42/p-T181-tau, γ-secretase
Tau targeted	Increase	t-tau, p-T181-tau, p-T231-tau, p-S202-tau, p-S396-tau, p-tau/t-tau, p-T181-tau/t-tau,-tau/Aβ42
	Decrease	t-tau
	No change	t-tau, p-T181-tau, p-S396-tau, p-T181-tau/t-tau, t-tau/Aβ42, N-224 tau, N-123 tau, MR tau, FL tau
Synaptic protein	Decrease	NRGN, synaptophysin, synaptotagmin, synaptopodin, SNAP-25, AMPA4 receptor, NPTX2, NLGN1, NRXN2, p-S9-synapsin 1, synapsin 1, MOG
Autolysosomal	Increase	cathepsin D, LAMP-1
	No change	cathepsin D, LAMP-1
Growth/trophic	Increase	GDNF
	Decrease	GDNF, FGF-2, FGF-13, HGF, IGF-1
Brain insulin resistance	Increase	p-Y-IRS-1, p-S312-IRS-1, p-S312-IRS-1/p-Y-IRS-1
	Decrease	p-Y-IRS-1
	No change	t-IRS-1, p-Y-IRS-1, p-S312-IRS-1, p-S312-IRS-1/p-Y-IRS-1
Inflammatory related	Increase	MMP-9, TSPO
	No change	IL-6
Molecular chaperone	Increase	ubiquitinylated protein
	Decrease	HSP70
	No change	HSP70, ubiquitinylated protein
Transcriptional repressor	Decrease	REST
Cell type marker	Increase	GFAP, NF-Lch, NS-enolase
	Decrease	GFAP, GluSyn
	No change	GluSyn, NF-Lch, NS-enolase
Exosome marker	Decrease	CD81
	No change	CD81, TSG101
Other	Increase	GM1
	Decrease	GAP43, Septin-8
	No change	Septin-8

## Data Availability

The data that support the findings of this study are available from the corresponding author upon reasonable request.

## References

[1] Zetterberg H., Bendlin B.B. (2021). Biomarkers for Alzheimer’s disease—Preparing for a new era of disease-modifying therapies. Mol. Psychiatry.

[2] Zhu D., Montagne A., Zhao Z. (2021). Alzheimer’s pathogenic mechanisms and underlying sex difference. Cell. Mol. Life Sci..

[3] Khan T.K., Alkon D.L. (2015). Alzheimer’s Disease Cerebrospinal Fluid and Neuroimaging Biomarkers: Diagnostic Accuracy and Relationship to Drug Efficacy. J. Alzheimer’s Dis..

[4] Mattson M.P. (2004). Pathways towards and away from Alzheimer’s disease. Nature.

[5] Wang J., Gu B.J., Masters C.L., Wang Y.J. (2017). A systemic view of alzheimer diseaseinsights from amyloid-beta metabolism beyond the brain. Nat. Rev. Neurol..

[6] Zetterberg H. (2019). Blood-based biomarkers for Alzheimer’s disease—An update. J. Neurosci. Methods.

[7] Khan T.K., Alkon D.L. (2015). Peripheral biomarkers of Alzheimer’s disease. J. Alzheimer’s Dis..

[8] Bălașa A.F., Chircov C., Grumezescu A.M. (2020). Body Fluid Biomarkers for Alzheimer’s Disease—An Up-To-Date Overview. Biomedicines.

[9] Janigro D., Bailey D.M., Lehmann S., Badaut J., O’Flynn R., Hirtz C., Marchi N. (2021). Peripheral Blood and Salivary Biomarkers of Blood–Brain Barrier Permeability and Neuronal Damage: Clinical and Applied Concepts. Front. Neurol..

[10] Seol W., Kim H., Son I. (2020). Urinary Biomarkers for Neurodegenerative Diseases. Exp. Neurobiol..

[11] McGrowder D., Miller F., Vaz K., Nwokocha C., Wilson-Clarke C., Anderson-Cross M., Brown J., Anderson-Jackson L., Williams L., Latore L. (2021). Cerebrospinal Fluid Biomarkers of Alzheimer’s Disease: Current Evidence and Future Perspectives. Brain Sci..

[12] Vichianin Y., Khummongkol A., Chiewvit P., Raksthaput A., Chaichanettee S., Aoonkaew N., Senanarong V. (2021). Accuracy of support-vector machines for diagnosis of alzheimer’s disease, using volume of brain obtained by structural mri at siriraj hospital. Front. Neurol..

[13] Bao W., Xie F., Zuo C., Guan Y., Huang Y.H. (2021). Pet neuroimaging of Alzheimer’s disease: Radiotracers and their utility in clinical research. Front. Aging Neurosci..

[14] Blennow K. (2017). A Review of Fluid Biomarkers for Alzheimer’s Disease: Moving from CSF to Blood. Neurol. Ther..

[15] Paraskevaidi M., Allsop D., Karim S., Martin F.L., Crean S. (2020). Diagnostic Biomarkers for Alzheimer’s Disease Using Non-Invasive Specimens. J. Clin. Med..

[16] Counts S.E., Ikonomovic M.D., Mercado N., Vega I.E., Mufson E.J. (2017). Biomarkers for the Early Detection and Progression of Alzheimer’s Disease. Neurotherapeutics.

[17] Song Z., Xu Y., Deng W., Zhang L., Zhu H., Yu P., Qu Y., Zhao W., Han Y., Qin C. (2020). Brain Derived Exosomes Are a Double-Edged Sword in Alzheimer’s Disease. Front. Mol. Neurosci..

[18] Lizarraga-Valderrama L.R., Sheridan G.K. (2021). Extracellular vesicles and intercellular communication in the central nervous system. FEBS Lett..

[19] Abels E.R., Breakefield X.O. (2016). Introduction to Extracellular Vesicles: Biogenesis, RNA Cargo Selection, Content, Release, and Uptake. Cell. Mol. Neurobiol..

[20] Saheera S., Potnuri A.G., Krishnamurthy P. (2020). Nano-Vesicle (Mis)Communication in Senescence-Related Pathologies. Cells.

[21] Gassama Y., Favereaux A. (2021). Emerging Roles of Extracellular Vesicles in the Central Nervous System: Physiology, Pathology, and Therapeutic Perspectives. Front. Cell. Neurosci..

[22] Tricarico C., Clancy J., D’Souza-Schorey C. (2017). Biology and biogenesis of shed microvesicles. Small GTPases.

[23] Malloci M., Perdomo L., Veerasamy M., Andriantsitohaina R., Simard G., Martínez M.C. (2019). Extracellular Vesicles: Mechanisms in Human Health and Disease. Antioxid. Redox Signal..

[24] Hornung S., Dutta S., Bitan G. (2020). CNS-Derived Blood Exosomes as a Promising Source of Biomarkers: Opportunities and Challenges. Front. Mol. Neurosci..

[25] Zetterberg H., Burnham S.C. (2019). Blood-based molecular biomarkers for Alzheimer’s disease. Mol. Brain.

[26] Watson L.S., Hamlett E.D., Stone T.D., Sims-Robinson C. (2019). Neuronally derived extracellular vesicles: An emerging tool for understanding Alzheimer’s disease. Mol. Neurodegener..

[27] Sinha M.S., Ansell-Schultz A., Civitelli L., Hildesjö C., Larsson M., Lannfelt L., Ingelsson M., Hallbeck M. (2018). Alzheimer’s disease pathology propagation by exosomes containing toxic amyloid-beta oligomers. Acta Neuropathol..

[28] Fiandaca M.S., Kapogiannis D., Mapstone M., Boxer A., Eitan E., Schwartz J.B., Abner E.L., Petersen R.C., Federoff H.J., Miller B.L. (2015). Identification of preclinical alzheimer’s disease by a profile of pathogenic proteins in neurally derived blood exosomes: A case-control study. Alzheimer’s Dement. J. Alzheimer’s Assoc..

[29] Goetzl E.J., Boxer A., Schwartz J.B., Abner E.L., Petersen R.C., Miller B.L., Kapogiannis D. (2015). Altered lysosomal proteins in neural-derived plasma exosomes in preclinical Alzheimer disease. Neurology.

[30] Kapogiannis D., Boxer A., Schwartz J.B., Abner E.L., Biragyn A., Masharani U., Frassetto L., Petersen R.C., Miller B.L., Goetzl E.J. (2015). Dysfunctionally phosphorylated type 1 insulin receptor substrate in neural-derived blood exosomes of preclinical alzheimer’s disease. FASEB J..

[31] Abner E.L., Jicha G.A., Shaw L.M., Trojanowski J.Q., Goetzl E.J. (2016). Plasma neuronal exosomal levels of Alzheimer’s disease biomarkers in normal aging. Ann. Clin. Transl. Neurol..

[32] Goetzl E.J., Kapogiannis D., Schwartz J.B., Lobach I.V., Goetzl L., Abner E.L., Jicha G.A., Karydas A.M., Boxer A., Miller B.L. (2016). Decreased synaptic proteins in neuronal exosomes of frontotemporal dementia and Alzheimer’s disease. FASEB J..

[33] Goetzl E.J., Mustapic M., Kapogiannis D., Eitan E., Lobach I.V., Goetzl L., Schwartz J.B., Miller B.L. (2016). Cargo proteins of plasma astrocyte-derived exosomes in Alzheimer’s disease. FASEB J..

[34] Winston C.N., Goetzl E.J., Akers J.C., Carter B.S., Rockenstein E.M., Galasko D., Masliah E., Rissman R.A. (2016). Prediction of conversion from mild cognitive impairment to dementia with neuronally derived blood exosome protein profile. Alzheimer’s Dement. Diagn. Assess. Dis. Monit..

[35] Guix F.X., Corbett G.T., Cha D.J., Mustapic M., Liu W., Mengel D., Chen Z., Aikawa E., Young-Pearse T., Kapogiannis D. (2018). Detection of Aggregation-Competent Tau in Neuron-Derived Extracellular Vesicles. Int. J. Mol. Sci..

[36] Goetzl E.J., Abner E.L., Jicha G.A., Kapogiannis D., Schwartz J.B. (2018). Declining levels of functionally specialized synaptic proteins in plasma neuronal exosomes with progression of alzheimer’s disease. FASEB J..

[37] Jia L., Qiu Q., Zhang H., Chu L., Du Y., Zhang J., Zhou C., Liang F., Shi S., Wang S. (2019). Concordance between the assessment of Aβ42, T-tau, and P-T181-tau in peripheral blood neuronal-derived exosomes and cerebrospinal fluid. Alzheimer’s Dement..

[38] Agliardi C., Guerini F.R., Zanzottera M., Bianchi A., Nemni R., Clerici M. (2019). SNAP-25 in Serum Is Carried by Exosomes of Neuronal Origin and Is a Potential Biomarker of Alzheimer’s Disease. Mol. Neurobiol..

[39] Chanteloup G., Cordonnier M., Moreno-Ramos T., Pytel V., Matías-Guiu J., Gobbo J., Cabrera-Martín M.N., Gómez-Pinedo U., Garrido C., Matías-Guiu J.A. (2019). Exosomal HSP70 for Monitoring of Frontotemporal Dementia and Alzheimer’s Disease: Clinical and FDG-PET Correlation. J. Alzheimer’s Dis..

[40] Cicognola C., Brinkmalm G., Wahlgren J., Portelius E., Gobom J., Cullen N.C., Hansson O., Parnetti L., Constantinescu R., Wildsmith K. (2019). Novel tau fragments in cerebrospinal fluid: Relation to tangle pathology and cognitive decline in Alzheimer’s disease. Acta Neuropathol..

[41] Goetzl E.J., Nogueras-Ortiz C., Mustapic M., Mullins R., Abner E.L., Schwartz J.B., Kapogiannis D. (2019). Deficient neurotrophic factors of CSPG4-type neural cell exosomes in Alzheimer disease. FASEB J..

[42] Kapogiannis D., Mustapic M., Shardell M.D., Berkowitz S., Diehl T.C., Spangler R.D., Tran J., Lazaropoulos M.P., Chawla S., Gulyani S. (2019). Association of Extracellular Vesicle Biomarkers with Alzheimer Disease in the Baltimore Longitudinal Study of Aging. JAMA Neurol..

[43] Gu D., Liu F., Meng M., Zhang L., Gordon M.L., Wang Y., Cai L., Zhang N. (2020). Elevated matrix metalloproteinase-9 levels in neuronal extracellular vesicles in Alzheimer’s disease. Ann. Clin. Transl. Neurol..

[44] Jia L., Zhu M., Kong C., Pang Y., Zhang H., Qiu Q., Wei C., Tang Y., Wang Q., Li Y. (2020). Blood neuro-exosomal synaptic proteins predict Alzheimer’s disease at the asymptomatic stage. Alzheimer’s Dement..

[45] Nam E., Lee Y.-B., Moon C., Chang K.-A. (2020). Serum Tau Proteins as Potential Biomarkers for the Assessment of Alzheimer’s Disease Progression. Int. J. Mol. Sci..

[46] Perrotte M., Haddad M., Le Page A., Frost E.H., Fulöp T., Ramassamy C. (2020). Profile of pathogenic proteins in total circulating extracellular vesicles in mild cognitive impairment and during the progression of Alzheimer’s disease. Neurobiol. Aging.

[47] Picciolini S., Gualerzi A., Carlomagno C., Cabinio M., Sorrentino S., Baglio F., Bedoni M. (2021). An SPRi-based biosensor pilot study: Analysis of multiple circulating extracellular vesicles and hippocampal volume in Alzheimer’s disease. J. Pharm. Biomed. Anal..

[48] Omar S.H., Preddy J. (2020). Advantages and Pitfalls in Fluid Biomarkers for Diagnosis of Alzheimer’s Disease. J. Pers. Med..

[49] Jin Q., Wu P., Zhou X., Qian H., Xu W. (2021). Extracellular Vesicles: Novel Roles in Neurological Disorders. Stem Cells Int..

[50] Eren E., Hunt J.F.V., Shardell M., Chawla S., Tran J., Gu J., Vogt N.M., Johnson S.C., Bendlin B.B., Kapogiannis D. (2020). Extracellular vesicle biomarkers of alzheimer’s disease associated with sub-clinical cognitive decline in late middle age. Alzheimer’s Dement. J. Alzheimer’s Assoc..

[51] Soto-Rojas L., Pacheco-Herrero M., Martínez-Gómez P., Campa-Córdoba B., Apátiga-Pérez R., Villegas-Rojas M., Harrington C., de la Cruz F., Garcés-Ramírez L., Luna-Muñoz J. (2021). The Neurovascular Unit Dysfunction in Alzheimer’s Disease. Int. J. Mol. Sci..

[52] Sagare A.P., Bell R.D., Zlokovic B.V. (2013). Neurovascular defects and faulty amyloid-beta vascular clearance in Alzheimer’s disease. J. Alzheimer’s Dis..

[53] Yamazaki Y., Kanekiyo T. (2017). Blood-Brain Barrier Dysfunction and the Pathogenesis of Alzheimer’s Disease. Int. J. Mol. Sci..

[54] Koelsch G. (2017). BACE1 Function and Inhibition: Implications of Intervention in the Amyloid Pathway of Alzheimer’s Disease Pathology. Molecules.

[55] Breijyeh Z., Karaman R. (2020). Comprehensive Review on Alzheimer’s Disease: Causes and Treatment. Molecules.

[56] Armstrong R.A. (2009). The molecular biology of senile plaques and neurofibrillary tangles in alzheimer’s disease. Folia Neuropathol..

[57] Chen G.-F., Xu T.-H., Yan Y., Zhou Y.-R., Jiang Y., Melcher K., Xu H.E. (2017). Amyloid beta: Structure, biology and structure-based therapeutic development. Acta Pharmacol. Sin..

[58] Tabaton M., Piccini A. (2005). Role of water-soluble amyloid-beta in the pathogenesis of Alzheimer’s disease. Int. J. Exp. Pathol..

[59] Eshraghi M., Adlimoghaddam A., Mahmoodzadeh A., Sharifzad F., Yasavoli-Sharahi H., Lorzadeh S., Albensi B., Ghavami S. (2021). Alzheimer’s Disease Pathogenesis: Role of Autophagy and Mitophagy Focusing in Microglia. Int. J. Mol. Sci..

[60] Iqbal K., Liu F., Gong C.-X., Grundke-Iqbal I. (2010). Tau in Alzheimer Disease and Related Tauopathies. Curr. Alzheimer Res..

[61] Milà-Alomà M., Suárez-Calvet M., Molinuevo J.L. (2019). Latest advances in cerebrospinal fluid and blood biomarkers of Alzheimer’s disease. Ther. Adv. Neurol. Disord..

[62] Toombs J., Zetterberg H. (2020). In the blood: Biomarkers for amyloid pathology and neurodegeneration in Alzheimer’s disease. Brain Commun..

[63] Zetterberg H., Wilson D., Andreasson U., Minthon L., Blennow K., Randall J., Hansson O. (2013). Plasma tau levels in Alzheimer’s disease. Alzheimers Res. Ther..

[64] Xing W., Gao W., Lv X., Xu X., Zhang Z., Yan J., Mao G., Bu Z. (2021). The diagnostic value of exosome-derived biomarkers in Alzheimer’s disease and mild cognitive impairment: A meta-analysis. Front. Aging Neurosci..

[65] Rastogi S., Sharma V., Bharti P.S., Rani K., Modi G.P., Nikolajeff F., Kumar S. (2021). The Evolving Landscape of Exosomes in Neurodegenerative Diseases: Exosomes Characteristics and a Promising Role in Early Diagnosis. Int. J. Mol. Sci..

[66] Crotti A., Sait H.R., McAvoy K.M., Estrada K., Ergun A., Szak S., Marsh G., Jandreski L., Peterson M., Reynolds T.L. (2019). BIN1 favors the spreading of Tau via extracellular vesicles. Sci. Rep..

[67] Mullins R.J., Mustapic M., Goetzl E.J., Kapogiannis D. (2017). Exosomal biomarkers of brain insulin resistance associated with regional atrophy in Alzheimer’s disease. Human Brain Mapp..

[68] Cersosimo E., DeFronzo R.A. (2006). Insulin resistance and endothelial dysfunction: The road map to cardiovascular diseases. Diabetes Metab. Res. Rev..

[69] Mullins R., Diehl T.C., Chia C.W., Kapogiannis D. (2017). Insulin Resistance as a Link between Amyloid-Beta and Tau Pathologies in Alzheimer’s Disease. Front. Aging Neurosci..

[70] Talbot K., Wang H.-Y., Kazi H., Han L.-Y., Bakshi K.P., Stucky A., Fuino R.L., Kawaguchi K.R., Samoyedny A.J., Wilson R.S. (2012). Demonstrated brain insulin resistance in Alzheimer’s disease patients is associated with IGF-1 resistance, IRS-1 dysregulation, and cognitive decline. J. Clin. Investig..

[71] Boudesco C., Cause S., Jego G., Garrido C. (2018). Hsp70: A Cancer Target Inside and Outside the Cell. Methods Mol Biol..

[72] Evans C.G., Wisen S., Gestwicki J.E. (2006). Heat shock proteins 70 and 90 inhibit early stages of amyloid beta-(1-42) aggregation in vitro. J. Biol. Chem..

[73] Magrane J., Smith R.C., Walsh K., Querfurth H.W. (2004). Heat shock protein 70 participates in the neuroprotective response to intracellularly expressed beta-amyloid in neurons. J. Neurosci..

[74] Yuyama K., Igarashi Y. (2017). Exosomes as carriers of Alzheimer’s amyloid-ss. Front. Neurosci..

[75] Yuyama K., Sun H., Sakai S., Mitsutake S., Okada M., Tahara H., Furukawa J., Fujitani N., Shinohara Y., Igarashi Y. (2014). Decreased amyloid-beta pathologies by intracerebral loading of glycosphingolipid-enriched exosomes in alzheimer model mice. J. Biol. Chem..

[76] Yuyama K., Sun H., Mitsutake S., Igarashi Y. (2012). Sphingolipid-modulated exosome secretion promotes clearance of amyloid-beta by microglia. J. Biol. Chem..

[77] An K., Klyubin I., Kim Y., Jung J.H., Mably A.J., O’Dowd S.T., Lynch T., Kanmert D., Lemere C.A., Finan G.M. (2013). Exosomes neutralize synaptic-plasticity-disrupting activity of abeta assemblies in vivo. Mol. Brain.

[78] Wang J.K.T., Langfelder P., Horvath S., Palazzolo M.J. (2017). Exosomes and Homeostatic Synaptic Plasticity Are Linked to Each other and to Huntington’s, Parkinson’s, and Other Neurodegenerative Diseases by Database-Enabled Analyses of Comprehensively Curated Datasets. Front. Neurosci..

[79] Xu B., Zhang Y., Du X.-F., Li J., Zi H.-X., Bu J.-W., Yan Y., Han H., Du J.-L. (2017). Neurons secrete miR-132-containing exosomes to regulate brain vascular integrity. Cell Res..

[80] Kalani A., Tyagi A., Tyagi N. (2013). Exosomes: Mediators of Neurodegeneration, Neuroprotection and Therapeutics. Mol. Neurobiol..

[81] Kalani A., Tyagi N. (2015). Exosomes in neurological disease, neuroprotection, repair and therapeutics: Problems and perspectives. Neural Regen. Res..

[82] Wang S., Cesca F., Loers G., Schweizer M., Buck F., Benfenati F., Schachner M., Kleene R. (2011). Synapsin I Is an Oligomannose-Carrying Glycoprotein, Acts As an Oligomannose-Binding Lectin, and Promotes Neurite Outgrowth and Neuronal Survival When Released via Glia-Derived Exosomes. J. Neurosci..

[83] Long X., Yao X., Jiang Q., Yang Y., He X., Tian W., Zhao K., Zhang H. (2020). Astrocyte-derived exosomes enriched with miR-873a-5p inhibit neuroinflammation via microglia phenotype modulation after traumatic brain injury. J. Neuroinflamm..

[84] Yang L., Niu F., Yao H., Liao K., Chen X., Kook Y., Ma R., Hu G., Buch S. (2018). Exosomal miR-9 Released from HIV Tat Stimulated Astrocytes Mediates Microglial Migration. J. Neuroimmune Pharmacol..

[85] Luarte A., Cisternas P., Caviedes A., Batiz L.F., Lafourcade C., Wyneken U., Henzi R. (2017). Astrocytes at the Hub of the Stress Response: Potential Modulation of Neurogenesis by miRNAs in Astrocyte-Derived Exosomes. Stem Cells Int..

[86] Bakhti M., Winter C., Simons M. (2011). Inhibition of Myelin Membrane Sheath Formation by Oligodendrocyte-derived Exosome-like Vesicles. J. Biol. Chem..

[87] Frühbeis C., Fröhlich D., Kuo W.P., Amphornrat J., Thilemann S., Saab A.S., Kirchhoff F., Möbius W., Goebbels S., Nave K.-A. (2013). Neurotransmitter-Triggered Transfer of Exosomes Mediates Oligodendrocyte–Neuron Communication. PLoS Biol..

[88] Gao G., Zhao S., Xia X., Li C., Li C., Ji C., Sheng S., Tang Y., Zhu J., Wang Y. (2019). Glutaminase C Regulates Microglial Activation and Pro-inflammatory Exosome Release: Relevance to the Pathogenesis of Alzheimer’s Disease. Front. Cell. Neurosci..

[89] Huang S., Ge X., Yu J., Han Z., Yin Z., Li Y., Chen F., Wang H., Zhang J., Lei P. (2018). Increased mir-124-3p in microglial exosomes following traumatic brain injury inhibits neuronal inflammation and contributes to neurite outgrowth via their transfer into neurons. FASEB J..

[90] Reynolds J.L., Mahajan S.D. (2019). Transmigration of Tetraspanin 2 (Tspan2) siRNA Via Microglia Derived Exosomes across the Blood Brain Barrier Modifies the Production of Immune Mediators by Microglia Cells. J. Neuroimmune Pharmacol..

[91] Yin Q.Q., Ji X., Lv R., Pei J.-J., Du Y., Shen C., Hou X. (2020). Targetting Exosomes as a New Biomarker and Therapeutic Approach for Alzheimer’s Disease. Clin. Interv. Aging.

